# TSPAN32 suppresses chronic myeloid leukemia pathogenesis and progression by stabilizing PTEN

**DOI:** 10.1038/s41392-022-01290-7

**Published:** 2023-03-01

**Authors:** Qiang Qiu, Yuanyuan Sun, Linyu Yang, Qingqing Li, Yunyu Feng, Mengyuan Li, Yuexia Yin, Li Zheng, Ning Li, Huandi Qiu, Xue Cui, Wei He, Bochuan Wang, Cong Pan, Zi Wang, Juan Huang, Klarke M. Sample, Zhihui Li, Yiguo Hu

**Affiliations:** 1 Department of Thyroid Surgery, National Clinical Research Center for Geriatrics, West China Hospital, Sichuan University, Chengdu, Sichuan China; 2 State Key Laboratory of Biotherapy and Cancer Center, West China Hospital, Sichuan University, and Collaborative Innovation Center for Biotherapy, Chengdu, Sichuan China; 3 Department of Oncology, Guizhou Provincial People’s Hospital, Guiyang, Guizhou China; 4 Sichuan Provincial People’s Hospital, Chengdu, Sichuan China; 5 Institute of Life Science, eBond Pharmaceutical Technology Ltd., Chengdu, China; 6 Laboratory of thyroid and parathyroid disease, Frontiers Science Center for Disease-related Molecular Network, West China Hospital, Sichuan University, Chengdu, Sichuan China; 7 Department of Thyroid Surgery, West China Hospital, Sichuan University, Chengdu, Sichuan China

**Keywords:** Haematological cancer, Haematopoietic stem cells

## Abstract

We report herein that *TSPAN32* is a key node factor for Philadelphia (Ph^+^) leukemia pathogenesis. We found that *TSPAN32* expression was repressed by BCR-ABL and ectopic TSPAN32 expression upon Imatinib treatment inhibited the proliferation of Ph^+^ cell lines. *Tspan32* overexpression significantly prevented BCR-ABL induced leukemia progression in a murine model and impaired leukemia stem cell (LSC) proliferation. LSCs represent an obstacle for chronic myeloid leukemia (CML) elimination, which continually replenish leukemia cells and are associated with disease relapse. Therefore, the identification of essential targets that contribute to the survival and self-renewal of LSCs is important for novel curative CML. Mechanistically, TSPAN32 was shown to interact with PTEN, increased its protein level and caused a reduction in PI3K-AKT signaling activity. We also found that *TSPAN32 was* repressed by BCR-ABL via the suppression of an important transcription factor, *TAL1*. Ectopic expression of TAL1 significantly increased *TSPAN32* mRNA and protein level, which indicated that BCR-ABL repressed *TSPAN32* transcription by decreasing *TAL1* expression. Overall, we identified a new signaling axis composed of “BCR-ABL-TAL1-TSPAN32-PTEN-PI3K-AKT”. Our findings further complement the known mechanisms underlying the transformation potential of BCR-ABL in CML pathogenesis. This new signaling axis also provides a potential means to target PI3K-AKT for CML treatment.

## Introduction

Chronic myeloid leukemia (CML) is a hematological malignancy, of which approximately 90% are caused by *BCR-ABL*. This reciprocal chromosomal translocation event, which is generated by t(9,22) (q34;q11), also contributes to between 20 and 30% of adult B cell acute lymphoid leukemia (B-ALL).^1^ *BCR-ABL* encodes the BCR-ABL oncoprotein which has sustained kinase activity, and is the central factor in initiating Ph^+^ leukemias and driving their progression. Accumulated evidence has shown that oncogenic transformation of hematopoietic cells by BCR-ABL relies upon both its kinase and non-kinase activity.^2^ BCR-ABL activates multiple signaling pathways upon activation of its kinase activity, including PI3K-AKT, JAK/STAT, RAS/RAF/MEK/ERK, MYC and WNT/CTNNB1.^3^ Some genes, such as *Alox5*, *Alox15*, *Scd1*, and *Blk*,^4,5^ are essential for CML stem cells (LSC) survival and are activated by BCR-ABL independent of its kinase activity. Clinically, BCR-ABL tyrosine kinase inhibitors (TKIs) have achieved great treatment outcomes for Ph^+^ leukemias.^6^ However, some patients do not respond well or relapsed due to BCR-ABL mutation (e.g., T315I, G250E, Q252H, Y253H) associated TKI resistance^7^ and other unknown reasons.^8^ CML blast crisis (BC) can occur with disease progression due to the accumulation of additional oncogenic hits, genomic instability, epigenetic changes, or microenvironmental shifts. BC and Ph^+^ B-ALL both respond poorly to TKI treatment.^1,9^ It is worth noting that LSCs are not sensitive to TKIs (when compared to mature leukemia cells), and contribute to the continual leukemia cells replenishment and respond to disease relapse.^4^ Therefore, the complete elimination of LSCs could provide a more optimal curative therapy for CML.^4,5^ There is still a need to develop new therapeutic strategies (and identify potential targets) to effectively restrain LSCs or overcome TKIs resistance.

Previous studies on BCR-ABL signaling have focused on intracellular molecules, such as non-receptor kinases, enzymes, transcription factors and adapter proteins, but less studies have reported on its associated membrane proteins.^10^ Accumulating evidence has shown that tetraspanin (TSPAN) proteins correlate with multiple cancer hallmarks, including cancer metastasis, proliferation, adhesion, stemness and chemotherapy resistance.^11,12^ For instance, TSPAN29 is involved in acute myeloid leukemia (AML) pathogenesis and drug resistance.^13^ Loss of TSPAN28 promotes AML chemosensitivity and disrupts leukemia cell bone marrow homing.^14^ TSPAN27 enhances AML survival by promoting IL10 secreting leukemia cells.^15^ TSPAN30 acts as an essential gene for the maintenance of hematopoietic stem cell (HSC) quiescence by supporting TGFB signaling.^16^

In this study, we demonstrated that TSPAN32 was a key downstream target of BCR-ABL involved in CML pathogenesis via PTEN protein stabilization. TSPAN32 is a member of the TSPAN superfamily, with four transmembrane regions consisting of the amino and carboxyl termini of the cytoplasm and two extracellular domains.^17^ Previous studies on *TSPAN32* have focused upon its regulation of immune system functions, especially with regard to T-cell regulation. *Tspan32* deficiency increases IL2 secretion and promotes T-cell proliferation, but significantly diminishes the ability to generate pathogen-specific T cells.^18^ *TSPAN32* expression is significantly reduced in memory T cells from patients with multiple sclerosis,^19^ and in activated B cells and plasma cells in systemic lupus erythematosus patients.^20^ These studies suggest that *TSPAN32* plays an important role in autoimmune diseases with potential to become a diagnostic marker or therapeutic target. However, the role of *TSPAN32* in Ph^+^ leukemias has not yet been explored.

Herein, scRNA-seq data for LSCs from CML patients was analyzed, which enabled the identification that *TSPAN32* expression was repressed by BCR-ABL. Ectopic expression of TSPAN32 in Ph^+^ cell lines inhibited cellular viability and increased the anti-tumor effects of Imatinib. Through the use of Tspan32 knock-in mice, it was shown that Tspan32 over-expression markedly prolonged the survival of BCR-ABL induced CML and B-ALL disease mice. Whereas LSC proliferation was impeded via the Tspan32 over-expression. Mechanically, TSPAN32 was revealed to interact with PTEN and increase its protein level, resulting in the downregulation of PI3K-AKT signaling activation. Additionally, BCR-ABL repressed *TSPAN32* transcription via *TAL1* expression suppression, a transcription factor regulating *TSPAN32* transcription. Overall, we revealed that the “TAL1-TSPAN32-PTEN” signaling axis played a negative regulatory role in Ph^+^ leukemias.

## Results

### BCR-ABL represses *TSPAN32* expression

**Fig. 1:**
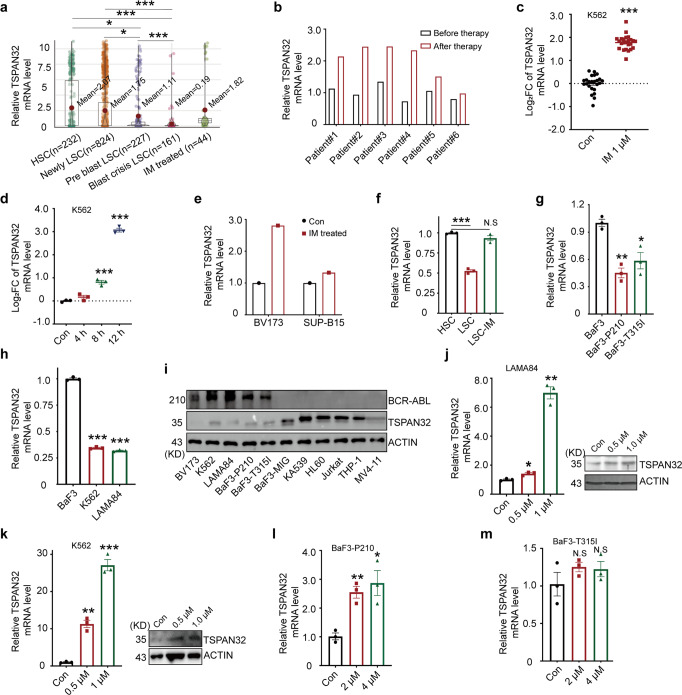
BCR-ABL decreases *TSPAN32* expression. **a** The GSE76312 scRNA-seq data for *TPSAN32* in CD34+ cells from healthy donors, BCR-ABL+CD34+ cells from different stages of CML patients, and CML patients treated with Imatinib (Healthy HSC, *n* = 232; Newly LSC, *n* = 824; Pre blast LSC, *n* = 227; Blast crisis LSC, *n* = 161; Imatinib treated, *n* = 44). **b** GSE12211 microarray data for *TSPAN32* expression in CD34+ cells from patients with chronic stage CML that received Imatinib. **c** GSE1922 microarray data for *TSPAN32* in K562 cells treated with 1 μM Imatinib for 24 h. **d** GSE51083 microarray data for *TSPAN32* in K562 cells treated with 100 nM Dasatinib for 4, 8 and 12 h. **e** An analysis of expression changes in microarray data (GES7182) for Ph^+^ B-ALL cell lines, which received 10 μM Imatinib for 16 h. **f** *Tspan32* mRNA was examined with RT-PCR in HSCs isolated from healthy C57/B6J mice, and LSCs were isolated from BCR-ABL induced CML recipients treated with or without Imatinib (*n* = 3, each group). **g** BaF3 cells transfected with BCR-ABL or BCR-ABL(T315I), RT-PCR analysis for *Tspan32* mRNA, BaF3 cells were used as the control. **h** RT-PCR analysis of TSPAN32 mRNA levels in BaF3, K562 and LAMA84 cells. **i** Protein levels of TSPAN32 in Ph^+^ cell lines (BV173, K562 and LAMA84), BaF3 cells transfected with BCR-ABL (P210 or T315I) or empty vector (MIG), and other hematologic malignancy cell lines without BCR- ABL fusion gene (KA539, HL60, Jurkat, THP-1, and MV4-11) were detected with immunoblotting. The expression statuses of BCR-ABL were also examined and shown. ACTIN served as sample loading control. **j**, **k** LAMA84 and K562 cells were treated with the indicated concentration of Imatinib for 24 h, the TSPAN32 protein and mRNA changes were analyzed with immunoblotting and RT-PCR, respectively. **l**, **m** BaF3 cells transfected with BCR-ABL or BCR-ABL(T315I) were treated with Imatinib at the indicated concentration for 24 h, and *Tspan32* mRNA was analyzed using RT-PCR. Error bars denote mean ± SEM, *P < 0.05, **P < 0.01, ***P < 0.001 (*T*-test)

By analyzing CML patient scRNA-seq data obtained from public datasets, we found that the expression of *TSPAN32* was decreased in the LSCs of newly diagnosed CML patients compared the HSCs from healthy donors. Its expression was further reduced with the disease progression, while its expression was significantly increased in the patients receiving Imatinib treatment (Fig. 1a). Furthermore, an analysis of bulk RNA-seq data for LSCs isolated from CML patients also showed that *TSPAN32* expression was increased after Imatinib exposure (Fig. 1b). To further confirm this observation, the expression of *TSPAN32* was analyzed in three different Ph^+^ cell lines treated with Imatinib or Dasatinib at different concentrations or with different exposure times with a fixed concentration. *TSPAN32 mRNA* was markedly increased under these conditions (Fig. 1c–e). To investigate whether *Tspan32* expression was decreased by BCR-ABL in LSCs, we examined *Tspan32* mRNA in mouse LSCs and HSCs and found that *Tspan32* mRNA was decreased in LSCs; while it was increased upon Imatinib exposure (Fig. 1f). Our in vitro data also supported this observation. In BCR-ABL (including T315I mutation) transformed BaF3 cells, the *Tspan32* mRNA was decreased by BCR-ABL (Fig. 1g). Additionally, the mRNA and protein levels of *Tspan32* were examined via RT-PCR and immunoblotting. Consistent with mRNA level results (Fig. 1h), the protein levels were lower in Ph^+^ cell lines and BCR-ABL transformed BaF3 cells (Fig. 1i). Moreover, compared to Ph^+^ cell lines, immunoblotting also showed that the TSPAN32 protein levels were higher in multiple hematologic malignancy cell lines without BCR-ABL fusion gene (Fig. 1i). Interestingly, both mRNA and protein were restored when BCR-ABL kinase activity was inhibited with Imatinib (Fig. 1j, k). When Imatinib was used to treat BCR-ABL and BCR-ABL(T315I) transformed BaF3 cells, we found that the expression of *Tspan32* was only restored in BCR-ABL transformed cells, but not in BCR-ABL(T315I) transformed cells (Fig. 1l, m). Together, these results indicated that *TSPAN32* was repressed by the kinase dependent activity of BCR-ABL.

### Ectopic expression of TSPAN32 inhibits Ph^+^ leukemia cell growth

**Fig. 2:**
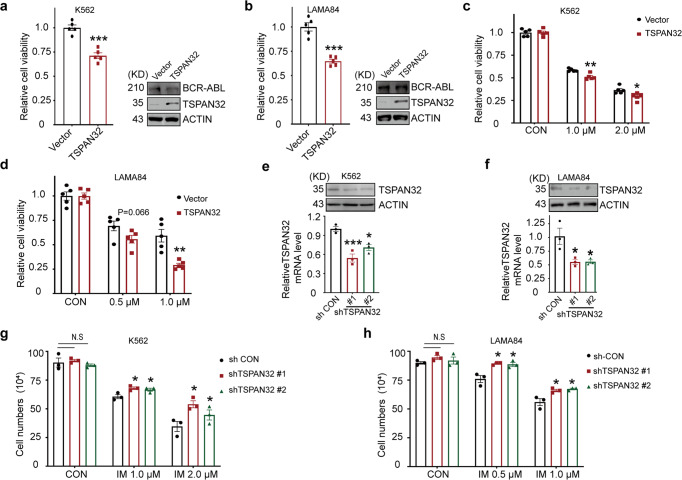
Overexpression of TSPAN32 synergizes with Imatinib for the inhibition of CML cell line growth. **a**, **b** K562 and LAMA84 cells with or without ectopic expression of TSPAN32 were seeded in 96 well plates for 48 h. Cell viability was measured with CCK8. Relative cell viability was normalized to control cells. The expression statuses of TSPAN32 and BCR-ABL were also examined and shown. ACTIN served as sample loading control. **c**, **d** K562 and LAMA84 cells with or without ectopic TSPAN32 expression were seeded in 96 well plates and treated with Imatinib at indicated concentration for 48 h. Cell viability was measured with CCK8. Relative cell viability was standardized with respective control cells. **e**, **f** The knockdown efficiency of shRNA targeting TSPAN32 in K562 and LAMA84 cells were examined with RT-PCR and immunoblotting. **g**, **h** K562 and LAMA84 cells with or without shRNA targeting TSPAN32 were plated and treated with Imatinib at the indicated concentration for 48 h, living cells were counted. Error bars denote mean ± SEM, *P < 0.05, **P < 0.01, ***P < 0.001 (*T*-test)

To investigate the role of *TSPAN32* in Ph^+^ leukemia cells, TSPAN32 was over expressed in K562 and LAMA84 cells via a lentiviral transduction. The ectopic expression of TSPAN32 inhibited the proliferation of these Ph^+^ leukemia cells (Fig. 2a, b). To exclude whether ectopic TSPAN32 expression decreased BCR-ABL expression, K562 and LAMA84 cells were transduced with TSPAN32 or an empty retroviral vector, and BCR-ABL protein levels were examined using western-blotting with an anti-ABL antibody. We found that ectopic TSPAN32 expression of did not suppress BCR-ABL expression (Fig. 2a, b). To investigate whether high TSPAN32 expression promotes the sensitivity of Ph^+^ leukemia cells to TKI induced cell death, K562 and LAMA84 cells were transfected with empty vector or TSPAN32 cDNA, and treated with Imatinib for 48 h. We observed that TSPAN32 overexpression in leukemia cells resulted in increased sensitivity to imatinib (Fig. 2c, d). Conversely, the use of shRNA to knockdown TSPAN32 in Ph^+^ leukemia cells (Fig. 2e, f), made leukemia cells less sensitive to Imatinib (Fig. 2g, h). Collectively, these results indicate that restoring TSPAN32 expression restrained Ph^+^ leukemia cell proliferation and promoted cell sensitivity to Imatinib.

### Tspan32 prevents the progression of BCR-ABL induced CML

A BCR-ABL induced CML mouse model was employed to investigate whether Tspan32 also plays a role in the suppression of CML in vivo. A single vector containing BCR-ABL and Tspan32 was constructed, and the vector was validated using western-blotting (Supplementary Fig. 1a). Then the virus was generated and the titer was determined (Supplementary Fig. 1b). The BCR-ABL induced CML model demonstrated that the disease progression was significantly slower for the BCR-ABL-Tspan32 transduced BM cell recipients, which was reflected by longer survival times (Supplementary Fig. 1c) and a lower leukemia cell burden in the examined organs (Supplementary Fig. 1d) when compared to that of mice receiving cells with BCR-ABL alone. Moreover, we found that there was a LSC lower percentage and cell number in the BM of the BCR-ABL-Tspan32 group recipients (Supplementary Fig. 1e). To further validate the disease transference capacity of the LSCs, a secondary BMT was performed, which was consistent with the primary BMT results. High expression of Tspan32 prevented leukemia progression and inhibited leukemia cell infiltration into peripheral organs, including the spleen and lung (Supplementary Fig. 1f, g). LSC analysis and statistics indicated that high Tspan32 expression significantly inhibited LSC percentage and number in disease mouse BM (Supplementary Fig. 1h). These results indicated that Tspan32 plays an important role in CML pathogenesis in the mouse model.

**Fig. 3:**
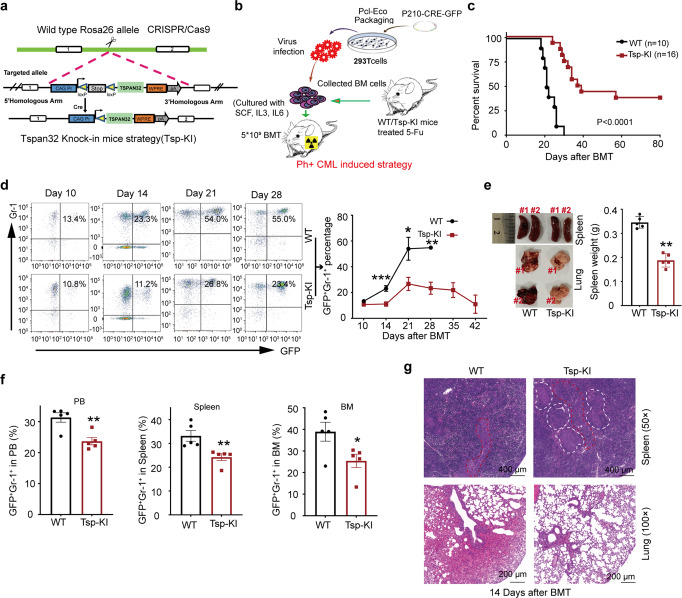
Overexpression of Tspan32 hampers the progression of BCR-ABL induced CML. **a** Schematic diagram describing the Tspan32 knock-in mouse with the Roas26 allele. **b** Schematic diagram describing Tspan32 overexpression via Cre endonuclease in a P210-iCre-GFP induced CML mouse model. **c** Kaplan–Meier-style survival curves for WT or Tsp-KI group recipients. **d** GFP+Gr-1+ percentages in PB for all recipients induced (day 10, 14, 28 and 32 post BMT). CML mice were induced, recipients from both groups were sacrificed on day 14 post BMT for the leukemia burden analysis (and infiltration in spleen and BM). **e** FACS analysis percentage for GFP+Gr-1+ cells in PB, Spleen and BM (*n* = 5 per group). **f** Gross appearances of lung and spleen, and spleen weight. **g** Photomicrographs for H&E-stained of spleen and lung sections. The white dotted line represents the white pulp of spleen, and red dotted line represents the red pulp of spleen. Error bars denote mean ± SEM, *P < 0.05, **P < 0.01, ***P < 0.001 (*T*-test)

The protein density of BCR-ABL expressed in BCR-ABL-Tspan32 co-expression vector was much lower than that expressed by BCR-ABL alone vector (Supplementary Fig. 1a), which may result in the lesser capabilities of BCR-ABL in disease transformation and progression. To overcome this problem, a mouse model with controllable Tspan32 expression (B6.Tspan32^T-loxp/0^) was produced (Fig. 3a). In the BCR-ABL induced CML model, high expression of Tspan32 was acquired by introducing iCre endonuclease with BCR-ABL into donor cells. Therefore, BM cells from WT and B6.Tspan32^T-loxp/0^ mice were transduced with virus containing BCR-ABL-iCre-GFP elements and injected into lethally irradiated recipients via the tail vein (Fig. 3b). Recipients receiving WT BM cells died of CML within 5 weeks post BMT; whereas mice receiving B6.Tspan32^T-loxp/0^ BM cells had a longer survival time. Indeed, 37.5% (6/16) of the recipients survived until the experimental end point (Fig. 3c). To monitor disease progression, leukemia cells (GFP+Gr1+) in disease mouse PB were measured with FACS. We found that the percentage of leukemia cells was lower in B6.Tspan32^T-loxp/0^ group recipients, reaching a peak on day 21 post BMT before declining (Fig. 3d). Leukemia cells in the BM and spleen were also analyzed on day 14. The leukemia burden was also lower in B6.Tspan32^T-loxp/0^ group recipients (Fig. 3e), which was consistent with the observation in PB. The lungs and spleens of B6.Tspan32^T-loxp/0^ group recipients also showed lesser severe synonyms. The spleens had a smaller size and lighter weight. There were fewer dark spots in the lungs. Furthermore, there was less infiltration of leukemia cells into the lungs and spleens (Fig. 3f, g).

### High-expression of Tspan32 impairs LSC proliferation

**Fig. 4:**
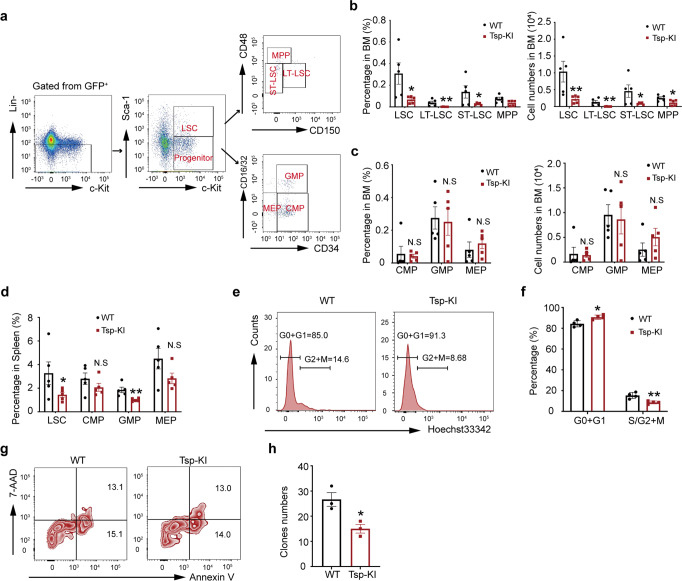
Tspan32 overexpression represses CML-LSCs proliferation and impedes its function. **a** On day 14 post BMT, recipient mice were analyzed to determine the percentage and number of LSCs and progenitors (*n* = 5 per group) (**b**) LT-LSCs, ST-LSCs and MPP percentage and number within LSCs in BM were analyzed (*n* = 5 per group). **c** CMP, GMP and MEP percentage and number within the progenitors in BM were analyzed (*n* = 5 per group). **d** The percentage of LSCs and progenitors in the spleen were analyzed (*n* = 5 per group). **e**, **f** The cell cycle analysis for BCR-ABL expressing LSCs from BM of recipient mice via Hoechst3342 staining (*n* = 4 per group). **g** BCR-ABL expressing LSC apoptosis within BM of recipient mice. **h** Colony formation assay. Sorted BCR-ABL-expressing LSCs from recipient mice BM were seeded into 6 well plates containing 2 mL M3234 medium, the total number of colonies were counted after 7 days, representative data displayed. Error bars denote mean ± SEM, N.S. no significance, *P < 0.05, **P < 0.01 (*T*-test)

High-expression of Tspan32 showed a suppressive role in LSCs (Supplementary Fig. 1e), which was further confirmed by examining LSCs and progenitors in BM on day 14 post BMT (Fig. 4a). We found that the LSC percentage and number (including LT-LSCs, ST-LSCs and MPPs) were dramatically lower in the B6.Tspan32^T-loxp/0^ group recipients (Fig. 4b). The progenitors (including CMPs and MEPs) in BM and spleens were not statistically different between the two groups (Fig. 4c, d). We further analyzed the cell cycle and viability of the LSCs and found that high-expression of Tspan32 resulted in a G0-G1 phase accumulation and a concomitant reduction in S + G2/M phase cells (Fig. 4e, f). This data suggests that Tspan32 blocked LSC cycle process. The LSCs viability analysis demonstrated that there was no statistical difference between apoptotic ratio of the two groups (Fig. 4g). An in vitro colony forming assay was conducted to investigate whether Tspan32 affects LSC self-renewal. The same amount of LSCs were isolated from BCR-ABL-iCre transduced WT or B6.Tspan32^T-loxp/0^ BM and seeded into stem cells culture medium. We found that B6.Tspan32^T-loxp/0^ LSCs generated significantly fewer colonies than WT LSCs, indicating that high Tspan32 expression impaired the self-renewal capability of LSCs (Fig. 4h).

### High-expression of Tspan32 does not affect hematopoiesis and HSC function

**Fig. 5:**
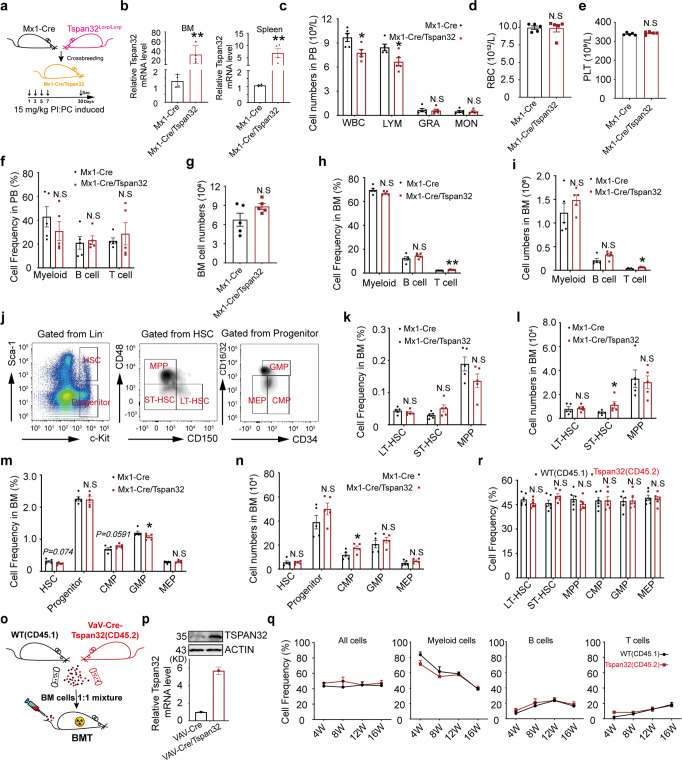
The effect of Tspan32 overexpression on the hematopoietic system in normal mice. **a**, **b** Mx1-Cre/Tsp-KI mice were treated four times with 15 mg/kg Pipc every other day. Tspan32 expression in cells from BM and spleen were analyzed with RT-PCR on day seven. Hematopoiesis and HSCs were analyzed on day 30 post Pipc induction (*n* = 5 per group). **c**–**e** Blood cell counts in the PB. **f** The percentage of lineage cells including myeloid cells (Gr-1), B cells and T cells in PB. **g** Total cell number counts in BM. (**h-i**) The percentage and number of lineage cells including myeloid cells (Gr-1), B cells and T cells in BM. **j** Analysis for HSC (LT-HSC, ST-HSC, MPP) and progenitors (CMP, GMP, MEP) according to cell surface markers. **k**, **l** The percentage and number of LT-HSC, ST-HSC and MPP in BM. **m**, **n** The percentage and number of HSCs and progenitors including CMP, GMP and MEP in BM. **o**, **p** Vav-Cre/Tsp-KI mice (CD45.2) mixture with CD45.1 BM cells at a 1:1 ratio for a competitive repopulation assay, RT-PCR and immunoblotting analysis for Tspan32 expression in cells from BM. **q** The percentage of replenished lineage cells in PB for recipients receiving an equal mix of BM cells with high Tspan32 expression (Cd45.2) and WT (Cd45.1) BM cells at weeks 4, 8, 12, and 16 post BMT. **r** The percentage of replenished HSCs (LT-HSC, ST-HSC, MPP) and progenitors (CMP, GMP, MEP) in the BM of recipients at week 16. Error bars denote mean ± SEM, N.S, no significance, *P < 0.05, **P < 0.01 (*T*-test)

To investigate whether high Tspan32 expression affected HSC function and hematopoiesis, B6.Tspan32^T-loxp/0^ mice were crossed with B6.Mx1-Cre mice to obtain mice with inducible Tspan32 expression (B6.Tspan32^T-loxp/0^Mx1-Cre) (Fig. 5a), in which Tspan32 can be specifically expressed by hematopoietic cells and hepatocytes after Pipc induction^21^ (Fig. 5b). Through this method we found that B6.Tspan32^T-loxp/0^Mx1-Cre mice had slightly lower numbers of white blood cells (WBCs) and lymphocytes (LYMs); similar counts of granulocytes (GRAs), monocytes (MONs), red blood cells (RBCs), and platelets in PB compared with that of WT mice (Fig. 5c–e). The percentage of myeloid and lymphocytes were not significantly different between the two groups (Fig. 5f). The percentage of myeloid cells, B cells and T cells were similar in the BM (Fig. 5h). The total cell number was slightly higher in the BM of B6.Tspan32^T-loxp/0^Mx1-Cre mice (Fig. 5g), where the T cell content was increased; but B cells and myeloid cells were not significantly different (Fig. 5i). The spleens from mice in both groups had similar weight and total cell counts (Supplementary Fig. 2a), and there were no significant differences in percentage and total cell count for B cells, T cells and myeloid cells (Supplementary Fig. 2b, c). Next, HSCs (LT-HSC, ST-HSC and MPP) and progenitor cells (CMP, GMP and MEP) in the BM of these mice were analyzed (Fig. 5j). We found that there were no significant differences between the WT and B6.Tspan32^T-loxp/0^Mx1-Cre mice for the LT-HSCs, ST-HSCs, MPPs, CMPs, GMPs and MEPs in terms of cell percentage and total cell count (Fig. 5k–n). The splenic analysis was similar to the BM analysis (Supplementary Fig. 2d, e). Thus, this data indicates that the high expression of Tspan32 had no obvious effects on HSCs and normal hematopoiesis.

To investigate whether high Tspan32 expression affected HSC function, a competitive repopulation assay was performed. To avoid the effects of Pipc on HSCs, B6.Tspan32^T-loxp/0^ mice were crossed with B6.Vav-Cre mice to obtain B6.Tspan32^T-loxp/0^Vav-Cre mice (Fig. 5o). The expression of Tspan32 was examined prior to the BMT as shown in Fig. 5p. The blood cells replenished from the high-Tspan32 donor (Cd45.2) and WT donor (Cd45.1) were examined with FACS at week 4, 8, 12, and 16 post BMT. We found that there were no obvious differences in the PB derived from Cd45.1 and Cd45.2 donors for the examined lineages, including myeloid cells (Gr1+), B cells (B220+) and T cells (CD3e+) (Fig. 5q). At week 16, HSCs and progenitors, including LT-HSC, ST-HSC, MPP, CMP, GMP and MEP in recipient BM were also analyzed, and no obvious differences were observed (Fig. 5r). Taken together, these results suggest that high-expression of Tspan32 does not affect the function of normal HSCs.

### High expression of Tspan32 prevents BCR-ABL induced B-ALL

**Fig. 6:**
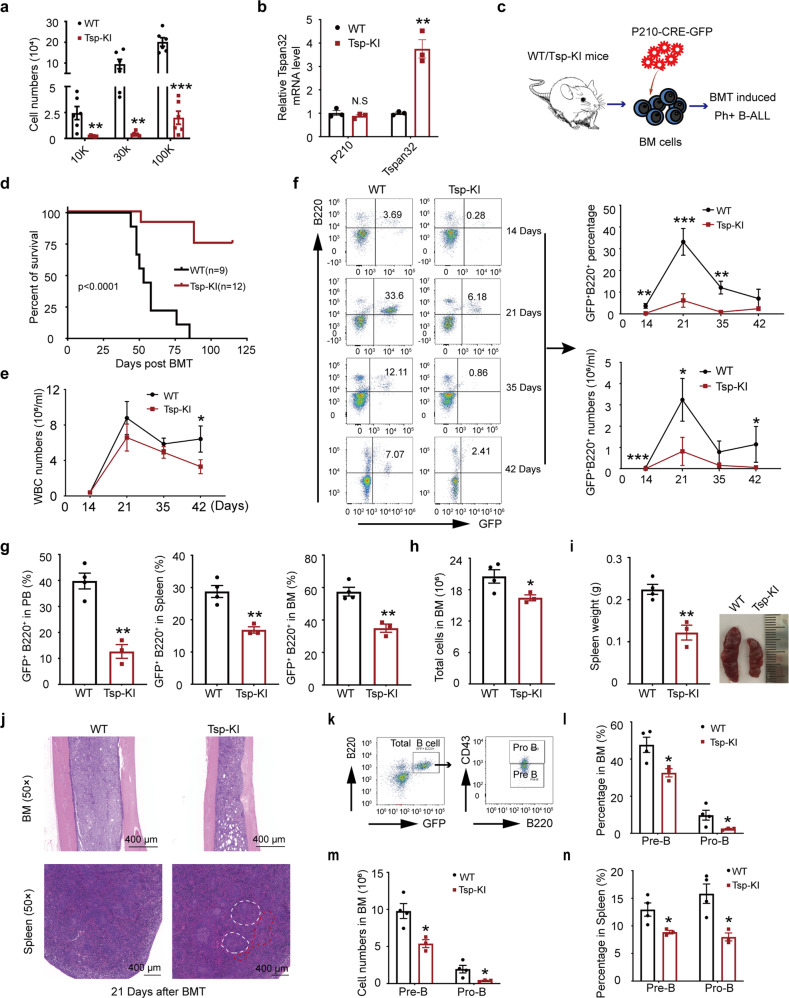
Overexpression of Tspan32 prevents the disease progression in BCR-ABL induced B-ALL. **a** Using WT and Tsp-KI mice as donors for Whitlock-Witte culture assay, total cell numbers were counted after 10 days, respectively. **b** BCR-ABL and Tspan32 mRNA were analyzed with RT-PCR. GFP served as the internal control. **c** Schematic diagram for the BCR-ABL(P210)-iCre-GFP induced B-ALL mouse model. **d** Kaplan–Meier-style survival curves for the WT or Tsp-KI group recipients. **e**, **f** The total white cell numbers and leukemia cell percentage were analyzed in the PB of all recipients on the indicated day post BMT. **g** The leukemia cell percentage in the PB, spleen and BM were analyzed on day 21 post BMT. **h**, **i** Total BM cell number, gross appearance and weight of spleen. **j** Photomicrographs for BM and spleen H&E staining from the recipients, the white dotted line represents the white pulp of spleen, and red dotted line represents the red pulp of spleen. **k**–**n** Pro-B (GFP+B220+CD43+) and pre-B (GFP+B220+CD43-) leukemia cells in BM and spleen were analyzed, and the percentage and total number were shown. (*n* = 3 per group), Error bars denote mean ± SEM, *P < 0.05, **P < 0.01, ***P < 0.001 (*T*-test)

Clinically, ~20–30% of adult ALL and 5% of pediatric ALL are driven by BCR-ABL.^22^ Firstly, we used public databases (GSE34861) to analyze the clinical correlation between TSPAN32 expression status and the survival of B-ALL patients. As shown in Supplementary Fig. 3a, BCR-ABL positive B-ALL patients with high TSPAN32 expression had a significantly higher probability of overall survival when compared to those with low TSPAN32 expression of. Thus, TSPAN32 expression status may serve as an independent predictor of clinical survival outcome for BCR-ABL positive B-ALL patients. To investigate the role of Tspan32 in Ph^+^ B-ALL, Whitlock-Witte culture was performed to test whether TSPAN32 plays a suppressive role in Ph^+^ B-ALL pathogenesis. We found that high Tspan32 expression significantly inhibited BCR-ABL transformed B cell growth (Fig. 6a, b). The biological function was subsequently evaluated in a BCR-ABL induced B-ALL mouse model (Fig. 6c). The recipients in the WT donor group died of B-ALL within 85 days post BMT; while of the recipient mice in the B6.Tspan32^T-loxp/0^ group, only 25% died of B-ALL by the end point (Fig. 6d). During the disease progression, we monitored the recipient leukemia burden from day 14 by counting leukemia cells in PB. We found that the total WBC numbers were significantly lower in B6.Tspan32^T-loxp/0^ group recipients compared to that in WT group recipients (Fig. 6e). The average percentage and number of leukemia cells (GFP+B220+) were significantly lower in the B6.Tspan32^T-loxp/0^ group recipients when compared to that in WT donor recipients (Fig. 6f). These results suggested that high Tspan2 expression impeded the progression of BCR-ABL induced B-ALL in vivo. Clinically, B cell lymphoid leukemia seriously infiltrates the BM and spleen, and affects normal hematopoiesis.^23^ Therefore, the BM and spleens for B-ALL mice were analyzed on day 21. The recipients were sacrificed and the distribution of leukemia cells in the PB, spleen and BM were analyzed for both groups. As shown in Fig. 6g, the leukemia cell percentages in the PB, BM and spleen of the B6.Tspan32^T-loxp/0^ group recipients were much lower when compared to that of the WT group recipients. Additionally, the infiltration of leukemia cells was much lower in the BM and spleens from the B6.Tspan32^T-loxp/0^ group recipients (Fig. 6h–j). Previous studies have demonstrated that B-ALL leukemia stem cells (B-LSCs) are enriched during GFP+B220+CD43+ population in BCR-ABL induced B-ALL mouse models and they could not be completely eliminated by Dasatinib.^24^ To test whether high-expression of Tspan32 also impedes B-LSCs, leukemia Pro-B and Pre-B cells were examined. We found that high Tspan32 expression notably decreased the percentage and number of Pro B and Pre-B cells both in the spleens and BM in the B6.Tspan32^T-loxp/0^ group recipients (Fig. 6k–n).

### TSPAN32 interacts with PTEN and increases its protein level and function

**Fig. 7:**
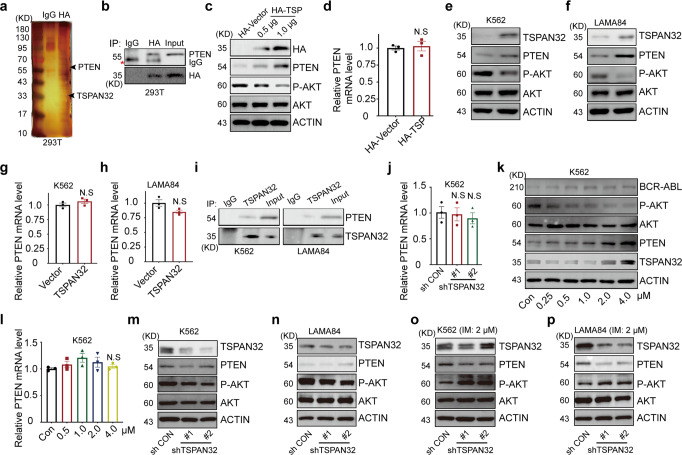
TSPAN32 interacts with PTEN protein and enhances its function. **a** The lysis of HEK-293T cells transfected with HA-TSPAN32 was performed for co-immunoprecipitation with the HA antibody or an IgG control, and the product was purified using a PAGE gel for mass spectrometry assay. **b** HEK-293T cell lysis was performed for co-transfected HA-TSPAN32 and PTEN for immunoprecipitation (with anti-HA and IgG) and immunoblotting (with anti-HA and anti-PTEN). **c**, **d** Indicated amount HA-TSPAN32 was transfected into HEK-293T cells for 24 h, RT-PCR for PTEN mRNA, and immunoblotting for TSPAN32, PTEN, P-AKT and total AKT, ACTIN served as the loading control. **e**–**h** K562 and LAMA84 with ectopic TSPAN32 expression; p-AKT, total AKT, PTEN and TSPAN32 were analyzed by immunoblotting, ACTIN served as the loading control, PTEN mRNA was analyzed by RT-PCR. **i** K562 and LAMA84 were treated with 2 μM Imatinib for 24 h, Immunoprecipitation (with anti-TSPAN32 and IgG) and immunoblotting analysis (with anti-TSPAN32 and anti-PTEN). **j** mRNA levels for PTEN were examined in K562 cells transfected with TSPAN32 shRNA. **k** Immunoblotting was performed for BCR-ABL, p-AKT, total AKT, PTEN and TSPAN32 in K562 cells treated with Imatinib at the indicated concentration for 24 h, ACTIN served as the loading control. **l** K562 cells treated with Imatinib at indicated concentration for 24 h, PTEN mRNA was analyzed with RT-PCR. **m**–**p** K562 and LAMA84 cells infected with TSPAN32 shRNA or control vector were treated with or without Imatinib for 24 h, immunoblotting was conducted for TSPAN32, PTEN, p-AKT, total AKT as indicated, ACTIN served as the loading control. N.S, no significance; error bars denote mean ± SEM

To elucidate the mechanism of TSPAN32 suppression in BCR-ABL transduced leukemias, the intracellular signaling transduction of TSPAN32 was investigated using a protein-protein interaction (PPI) network.^25^ To identify TSPAN32 binding partners, we overexpressed HA-TSPAN32 in 293 T cells, then performed immunoprecipitation with HA antibody for a peptide mass spectrometry (MS) assay (Fig. 7a). Based upon the MS data and the biological roles of TSPAN32 in Ph^+^ leukemias, PTEN was identified as a potential target for further studies. The interaction between PTEN and TSPAN32 was confirmed with a co-immunoprecipitation assay (Fig. 7b). To reveal the functional relationship between the two proteins, TSPAN32 cDNA was transfected into 293 T cells, where the PTEN protein gradually increased and synchronized with the level of TSPAN32. Whereas, the activity of AKT, which is a direct downstream target of PTEN, gradually decreased (Fig. 7c). Further studies revealed that the increased PTEN levels were not related to its mRNA expression (Fig. 7d), which indicated that TSPAN32 does not affect *PTEN* transcription and mRNA stability. Therefore, it is possible that TSPAN32 stabilizes PTEN protein. To test whether TSPAN32 also regulates PTEN during BCR-ABL driven conditions, we examined PTEN protein levels and activity in K562 and LAMA84 cell lines with ectopic TSPAN32 expression. We found that PTEN levels increased, with a corresponding p-AKT decrease (Fig. 7e, f). Like the 293 T cells, PTEN mRNA expression was comparable in the control and experimental lines (Fig. 7g, h). We also confirmed that TSPAN32 interacted with PTEN in Ph^+^ lines (K562 and LAMA84 cells), which indicated that the interaction was independent of BCR-ABL (Fig. 7i). Moreover, we found that decreasing TSPAN32 with shRNA (Fig. 2e) did not affect PTEN mRNA level (Fig. 7j) and PTEN protein levels were not notably different (Fig. 7m, n). We found that TSPAN32 and PTEN were increased when K562 cells were treated with Imatinib; while p-AKT was decreased (Fig. 7k), and PTEN mRNA did not change (Fig. 7l). Ph^+^ cell lines with control shRNA (shCON) or shRNAs targeting TSPAN32 were treated with Imatinib to examine whether the PTEN increase was related to TSPAN32. We found that PTEN protein level was lower in cells with shRNAs targeting TSPAN32, which correlated with higher p-AKT activity (Fig. 7o, p). These results suggest that the elevated PTEN protein levels caused by Imatinib is dependent upon the increase of TSPAN32.

### TAL1 regulates *TSPAN32* transcription

**Fig. 8:**
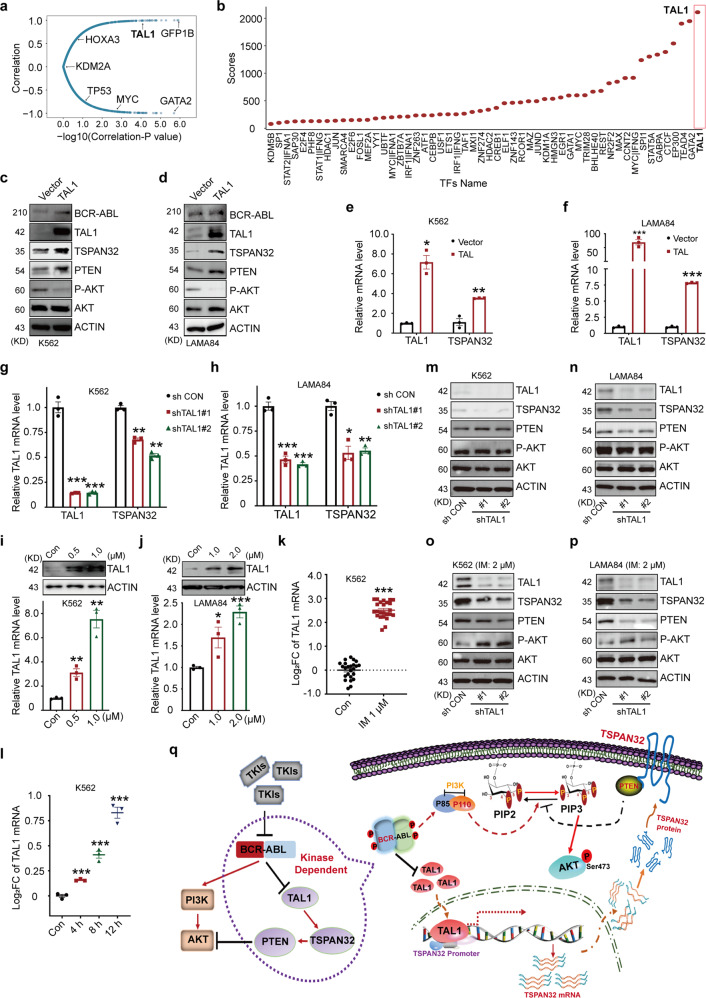
TAL1 is involved in the transcriptional regulation of TSPAN32. **a** An analysis for all transcription factors that are correlated with TSPAN32 expression in microarray data from K562 cells treated with 100 nM Dasatinib for 12 h (GSE51083). **b** Potential transcriptional factors were statistically ranked based upon *TSPAN32* expression from K562 CHIP-Seq data. **c**, **d** Immunoblotting was performed for BCR-ABL, TAL1, TSPAN32, PTEN, p-AKT and total AKT in K562 and LAMA84 cells with TAL1 ectopic expression, ACTIN served as loading control. **e**, **f** TAL1 and TSPAN32 mRNA was analyzed in K562 and LAMA84 cells with ectopic TAL1 expression. **g**, **h** Targeting of TAL1 mRNA with shRNA in K562 and LAMA84 cells, TAL1 and TSPAN32 mRNA were analyzed. **i**, **j** TAL1 protein and mRNA levels were analyzed by immunoblotting and RT-PCR in K562 and LAMA84 cells treated with Imatinib at the indicated concentration for 24 h. **k** *TAL1* expression in K562 cells treated with Imatinib for 24 h (GSE1922). **l** *TAL1* expression in K562 cells were treated with 100 nM Dasatinib for 4, 8 and 12 h (GSE51083). **m**–**p** Immunoblotting was performed for TAL1, TSPAN32, PTEN, p-AKT, total AKT, in K562 and LAMA84 cells with shRNA targeting TAL1 treated with or without Imatinib at the indicated concentration for 24 h; ACTIN served as loading control. **q** Working model for the “BCR-ABL-TAL1-TSPAN32-PTEN/PI3K-AKT” signaling axis in Ph^+^ leukemia. Error bars denote mean ± SEM, *P < 0.05, **P < 0.01, ***P < 0.001 (*T*-test)

Our previous studies demonstrated that BCR-ABL decreased the expression of *TSPAN32*. To reveal the detailed regulatory mechanisms underlying this process, a transcription factor analysis was conducted. Firstly, we systematically analyzed transcription factors that correlated with *TSPAN32* expression using transcriptome data from K562 cells treated with Imatinib (Fig. 8a) and ChIP-seq data for K562 cells from the SSP (The Signaling Pathways Project) database. All the potential transcription factors involved in *TSPAN32* regulation were scored (Fig. 8b). According to points of overlap between the two analyses, TAL1 was considered to be the candidate with the highest potential. To verify the hypothesis, we generated two stably transduced K562 and LAMA84 lines to constitutively express TAL1. When compared to the control cell lines, both TSPAN32 mRNA and protein were significantly increased in the cell lines with high TAL1 expression (Fig. 8c–f). On the other hand, shRNA mediated TAL1 knockdown resulted in a reduction of both TSPAN32 mRNA and protein (Fig. 8g, h, m). Next, we tested whether the increase of *TSPAN32* in Ph^+^ cell lines treated with Imatinib was due to *TAL1* transcriptional activity. First, K562 and LAMA84 cells were treated with Imatinib at different concentrations for 24 h. We found that both TAL1 mRNA and protein were significantly increased after Imatinib exposure (Fig. 8i, j), similar results were observed by analyzing K562 cell treated with imatinib or dasatinib from public database (Fig. 8k, l). When K562 and LAMA84 cells with shRNAs targeting TAL1 were treated with Imatinib, we found that *TSPAN32* expression was not elevated (Fig. 8o, p). Functionally, we demonstrated that TAL1 overexpression resulted in higher PTEN protein levels and lower AKT activity (Fig. 8c, d). These results indicated that *TSPAN32* expression was decreased by BCR-ABL via repressing *TAL1* expression. Lastly, we further analyzed the clinical correlation between the expression status of TAL1 and overall survival of Ph^+^ B-ALL patients from public database. Similar to TSPAN32, it was indicated that high expression level of TAL1 correlated strongly with higher overall survival probability (Supplementary Fig. 3b).

## Discussion

Although in the last two decades, Imatinib and other TKIs have achieved dramatic prognostic improvements for CML patients. The majority of patients that experience TKI resistance have point mutations within BCR-ABL. There are other unknown reasons for some Ph^+^ patients to develop resistance (or respond poorly) to TKI treatment despite the absence of these known mutations.^8^ Therefore, it is still important to further elucidate the mechanisms of TKI resistance and identify new therapeutic strategies for CML. Herein, we identified a new signaling axis “BCR-ABL-TAL1-TSPAN32-PTEN-PI3K-AKT” and reveal its biological roles in BCR-ABL induced leukemias and TKI therapy. Our findings complement the pathogenic mechanisms of BCR-ABL transformation and potentially provide a rationale strategy for targeting PI3K-AKT in CML.

In this study, we identified *TSPAN32* as a tumor suppressor gene in Ph^+^ leukemias, whose expression was decreased by BCR-ABL (Fig. 1). The biological roles of *TSPAN32* were not studied in Ph^+^ leukemias before. Previously, the suppression of *TSPAN32* by the AML-ETO fusion protein has been shown to involve in AML pathogenesis.^26^ When we ectopically expressed TSPAN32 in Ph^+^ cell lines, we found that TSPAN32 suppressed cell proliferation and increased TKI susceptibility (Fig. 2). Inversely, shRNA knockdown TSPAN32 impaired inhibition of Ph^+^ cell lines of IM. Interestingly, TSPAN32 knockdown alone did not show inhibitory effect on Ph^+^ cell lines (Fig. 2g, h). Previously studies have shown overlapping biological functions and complementary role among Tetraspanin family members,^27^ thus, we speculate that other tetraspanin members might compensate for the function loss of TSPAN32. Additionally, when we highly expressed TSPAN32 with BCR-ABL in a CML mouse model, we found that TSPAN32 significantly prevented CML progression (Supplementary Fig. 1). Technically, we found that the expression density of BCR-ABL was lower in BCR-ABL-TSPAN32 co-expressing vector in comparison to that in BCR-ABL alone vector (Supplementary Fig. 1a), which may lead to different capabilities of BCR-ABL in cell transformation and disease progression. To exclude the artificial bias, we generated a Tspan32 knock-in transgenic mouse model. By using transgenic mouse as donor, it was notably that high-expression of Tspan32 significantly prevented CML progression (Fig. 3). Clinically, the targeting and elimination of LSCs are essential for CML elimination.^4,5^ Strikingly, we found that high Tspan32 expression also suppressed LSC proliferation and impaired their function (Fig. 4), which suggested that targeting Tspan32 network might offer a potential strategy for LSC elimination. When we analyzed leukemias at different disease stages, some cell subpopulations had statistically significant differences; while other cell subpopulations with high Tspan32 expression displayed no difference when compared to the control groups (Fig. 4a–d). Clinically, it is believed that CML originates from LT-LSCs. The differentiation of downstream leukemia cells including ST-LSCs, MPPs, CMPs, GMPs, MEPs and granulocytes are replenished from LT-LSCs. Therefore, if the LSC percentage and number are lower in the B6.Tspan32^T-loxp/0^ group recipients, theoretically all the derived down-stream populations should be lower. The specific reason(s) for the differences observed herein are currently unknown. However, the most likely explanation is due to the fact that BM cells (including LT-HSCs, ST-HSCs, MPPs, CMPs, GMPs, and MEPs) were used as donors for the BCR-ABL transformation in the BCR-ABL induced CML model. Thus, some leukemia subpopulations in the diseased mice (that were analyzed on day 14 post BMT) are not totally derived from LT-HSCs, and other populations may also contribute via self-renewal and differentiation.

Compared to CML treatment, the current status of clinical treatment Ph^+^ B-ALL is not very optimistic. We also demonstrated that high-expression of Tspan32 significantly prevented BCR-ABL induced B-ALL and high expression levels of TSPAN32 correlated strongly with higher overall survival probability in Ph^+^ B-ALL patients (Fig. 6 and Supplementary Fig. 3a). Thus, TSPAN32 expression status may as a predictor of Ph^+^ B-ALL patients outcome. When comparing the effects of TSPAN32 on CML (Fig. 3c) and B-ALL (Fig. 6d), TSPAN32 may have a stronger suppressive effect on B-ALL than CML. It may be worth confirming these findings in the future and demonstrate the mechanism. Through the analysis of data from public database, we found that high TSPAN32 expression correlated strongly with a higher overall survival probability in Ph^+^ B-ALL patients (Supplementary Fig. 3a). Due to a lack of CML data, we could not evaluate the relationship between TSPAN32 expression and the overall survival for CML patients.

Mechanistically, we found that TSPAN32 interacted with and stabilized PTEN, resulting in PI3K-AKT pathway repression (Fig. 7). Multiple studies have reported the suppression of PTEN by BCR-ABL in CML,^28,29^ P53, EGR1 or MAPKK4 have been proposed to mediate this interaction at transcriptional level.^30–32^ Previous studies have also shown that PTEN deficiency promotes Ph^+^ CML and B-ALL development.^29,33^ It has been demonstrated that phosphorylation of PTEN at specific residues (Ser380, Thr382, Thr383 and Thr385) in its C-terminal tail is associated with increased stability, whereas phosphorylation at other sites decreases its stability.^34^ Recently, several E3 ubiquitin ligases and deubiquitylases have been reported to regulate PTEN stability via ubiquitination/deubiquitylation at lysine sites.^35–41^ Herein, we reported that TSPAN32 interacted with PTEN and increased its stability. It is possible that TSPAN32 might prevent PTEN from being phosphorylated or ubiquitinated by binding certain sites. Further studies may be warranted to further reveal the mechanisms underlying this interaction.

We further demonstrated that TAL1 was involved in the transcriptional regulation of *TSPAN32* (Fig. 8). TAL1 has an important role in hematopoiesis and leukemogenesis. TAL1 is essential for HSC retention in quiescence and stemness maintenance.^42^ TAL1 is also involved in the differentiation of myeloid and lymphoid linages.^43^ Approximately 60% of T-ALL is characterized by abnormal *TAL1* expression.^44^ Whereas, TAL1 is significantly decreased in CD34+ LSCs from CML patients, and loss of TAL1 inhibits Imatinib sensitive CML via induced apoptosis by reducing PTEN expression.^45^ Our data supports the notion that the inhibition of BCR-ABL kinase activity with Imatinib can cause elevated TSPAN32 protein levels, which stabilize PTEN and result in negative regulation of the PI3K-AKT pathway. The public database analysis identified that lower TAL1 expression correlated with poor overall survival probability for Ph^+^ B-ALL patients (Supplementary Fig. 3b).

*TSPAN32* is mostly expressed in the BM, spleen and thymus. It is known via *Tspan32* deficient mice that this gene plays an important role in T cell regulation.^18^ Moreover, decreased *TSPAN32* expression in lymphocytes may be associated with various autoimmune diseases.^19,20^ Herein, we found that high Tspan32 expression did not obviously affect hematopoiesis and HSC function in normal mice (Fig. 5 and Supplementary Fig. 2). Thus, it is possible that signaling alteration in normal cells caused by high Tspan32 expression could be overcome by self-regulation. Overall, we identified a new signaling axis “BCR-ABL-TAL1-TSPAN32-PTEN-PI3K-AKT” in Ph^+^ leukemias (Fig. 8q). During disease progression, genetic or epigenetic dysregulation might lead to insufficient expression of *TAL1* or *TSPAN32*, resulting in TKI resistance or a poor response.^46,47^ Thus, with further studies the targeting of the TSPAN32 regulatory network may be utilized to promote TKI response during CML treatment.

## Materials and methods

### Bioinformatics

The following publicly available GEO datasets were analyzed: GSE76312, GSE1922, GSE51083, GSE12211, GES7182 and GSE34861. GSE76312 is comprised of scRNA-seq data for CD34+ cells isolated from healthy donors, BCR-ABL+CD34+ cells isolated from CML different phases, and CML patients treated with TKIs. GSE1922 is microarray data of K562 cells treated with 1 μM Imatinib for 24 h. GSE51083 is a microarray data of K562 cells treated with 100 nM Dasatinib for 4, 8 and 12 h. GES7182 is microarray data of Ph^+^ B-ALL cell lines treated with 10 μM Imatinib for 16 h. GSE12211 is microarray data of CD34+ cells isolated from chronic phase CML patients receiving Imatinib therapy. GSE34861 is microarray data of adult B-lineage acute lymphoblastic leukemia (B-ALL) with Ph^+^ subtypes. All datasets were re-analyzed and the expression of TSPAN32 was determined using standard methods.

### DNA constructs

The oligo sequences targeting human TSPAN32 and TAL1 mRNA were obtained using the Broad Institute RNA interference platform (GPP Web Portal-Home) (miTSPAN32-1: 5′-TTGGACCGCAAGGGCAAATAC-3′, miTPAN32-2: 5′-CACTCCGAAGCAGTTGCTATT-3′, miTAL1-1: 5′-GCCCAGCATTTGGGTAATTTA-3′, miTAL1-2: 5′-ACCAAAGTTGTGCGGCGTATC-3′), which were cloned into the pLvx lentivirus vector with a puromycin resistance tag.

### Cell lines modifications and related assays

Ph^+^ leukemia cell lines (BV173, K562 and LAMA84) and Ph^-^ hematologic malignancy cell lines (KA539, HL60, Jurkat, THP-1, and MV4-11) were cultured with RPMI 1640 medium, 10% FBS (Gibco) and 100 units/ml penicillin/streptomycin (Gibco). The BaF3 cell cultures were supplemented with 50 μM 2-mercaptoethanol and 0.1 ng/mL mIL-3. Imatinib was purchased from MCE and dissolved in DMSO. BaF3-BCR-ABL (P210 or T315I) cells were generated by transducing BaF3 cells with retrovirus containing BCR-ABL (P210 or T315I).

K562 and LAMA84 cells were transduced with lentivirus containing *TSPAN32* or *TAL1*, or shRNAs targeting TSPAN32 or TAL1, the positive cells were selected using 2 μg/mL puromycin for 2 days, then 2 × 10^4^ cells were seeded into a 96 well plate 48 h prior to the cell viability assay, which was measured using CCK8 (relative to cells transduced with empty vector virus).

### Mice

All animal experiments were performed in accordance with guidelines approved by the Institutional Animal Care and Use Committees of Sichuan University, with protocols approved by the Animal Care and Use Committee of the State Key Laboratory of Biotherapy, Sichuan University. Recipient C57BL/6 (Cd45.2) mice purchased from Gempharma tech (Sichuan, CN) and maintained at the State Key laboratory of Biotherapy Animal Center. The B6.Mx1-Cre and B6.Vav-Cre mice were gifted by Cyagen (Jiangsu, China).

A tissue-specific Tspan32 inducible expression CRISPR/CAS9 transgenic model (Tspan32^T-loxp/0^) was generated using C57BL/6 (Cd45.2) background mice. Briefly, a loxp-flanked “stop” sequence was inserted between the CAG promoter and Tspan32 cDNA to block its translation, and a Roas26 allele element was knocked in. Then B6.Tspan32^T-loxp/0^ mice were crossed with B6.Mx1-Cre mice to obtain B6.Tspan32^T-loxp/0^Mx1-Cre mice. B6.Tspan32^T-loxp/0^Mx1-Cre mice were treated four times with poly(inosinic)-poly(cytidylic) acid (Pipc) at 15 mg/kg to induce Cre expression. The Cre recombinase removed the stop sequence resulting in Tspan32 expression. B6.Tspan32^T-loxp/0^Vav-Cre mice were obtained by crossing B6.Tspan32^T-loxp/0^ mice with B6.Vav-Cre mice.

### Analysis of HSCs and progenitors

B6.Tspan32^T-loxp/0^Mx1-Cre mice (8–10 weeks old) were treated four times with 15 mg/kg Pipc via an IP injection once every other day, and sacrificed for analysis at the indicated times. HSCs and progenitors were categorized using the following markers: HSCs: Lin^-^ Sca-1^+^ c-Kit^+^; LT-HSCs: Lin^-^ Sca-1^+^ c-Kit^+^CD48^-^CD150^+^; ST-HSCs: Lin^-^ Sca-1^+^ c-Kit^+^CD48^-^CD150^-^; MPPs: Lin^-^ Sca-1^+^ c-Kit^+^CD48^+^CD150^-^; CMPs: Lin^-^Sca-1^-^c-Kit^+^CD16/32^-^CD34^+^; GMPs: Lin^-^Sca-1^-^c-Kit^+^ CD16/32^+^CD34^+^; MEPs: Lin^-^ Sca-1^-^ c-Kit^+^ CD16/32^-^CD34^-^.

### BCR-ABL induced CML mouse model

Donor mice were pretreated with 200 mg/kg 5-fluorouracil (5-FU) via tail vein injection. Four days later, bone marrow (BM) cells were harvested from the femurs and tibias and cultured for 24 h with stimulated medium containing IL-3 (10 μg/mL), IL-6 (10 μg/mL) and SCF (50 μg/mL). The cells were subjected to two rounds of co-sedimentation/transduction with high-titer retroviral stock containing BCR-ABL at 1000Xg for 90 min. The transduced cells were subsequently transplanted into C57BL/6 J recipients (5 × 10^5^ cells per mouse) that had received two doses of 500 cGy of X-ray irradiation separated by 3 h. Leukemia cells (GFP+Gr-1+) in peripheral blood (PB), leukemia stem cells (LSCs: GFP^+^HSCs; LT-LSCs: GFP^+^LT-HSCs; ST-LSCs: GFP^+^ST-HSCs) and leukemia progenitors (GFP^+^MPPs, GFP^+^CMPs, GFP^+^GMPs, GFP^+^MEPs) in spleen or BM of recipients were analyzed at the indicated time post bone marrow transplantation (BMT).

### BCR-ABL induced B-ALL mouse model

Briefly, BM cells from donor femurs and tibias were transduced with retrovirus containing BCR-ABL. Recipients received two doses of 500 cGy of X-ray irradiation separated by 3 h. Then, 1 × 10^6^ transfected BM cells were transplanted into recipients via tail vein injection. Leukemia cells (GFP+B220+) in the PB, spleen and BM of recipients were analyzed at indicated time post BMT.

### Competitive repopulation assay

Equal BM cell numbers from B6.Tspan32^T-loxp/0^Vav-Cre (Cd45.2) and B6.SJL (Cd45.1) donor mice were mixed and transplanted into lethally irradiated B6 recipients at a dose of 1 × 10^6^ cells/per mouse. The repopulating capacity in PB was analyzed at week 4, 8, 12, and 16 post BMT. The percentages of donor derived HSC (LT-HSC, ST-HSC, MPP) and progenitors (CMP, GMP, MEP) in the recipient BM were analyzed in detail at 16 weeks post BMT.

### Whitlock-Witte cultures

The Whitlock-Witte culture assay was performed as previously described^48^ Briefly, BM cells from WT and B6.Tspan32^T-loxp/0^ mice tibias and femurs were transduced once with BCR-ABL-iCre-GFP retrovirus and cultured with lymphoid media (RPMI1640 plus 10% PBS, 1 U/ml penicillin, 1 μg/ml streptomycin, 1% L-glutamine and 50 μM 2-mercaptoethanol). Approximately 100 K, 30 K and 10k cells were seeded into a 24 well plate in triplicate, with a final cell number of 1 × 10^6^ by complementing with BM cells. B-lymphoid cells were counted 10 days post seeding.

### Flow cytometry

Cells were collected from the PB, spleen and BM of model mice. Red blood cells were lysed with RBC lysis buffer (pH7.4), washed twice with PBS and stained with following antibodies: APC-Gr1 for neutrophils; PE-CD3e for T cells; Pacific Blue-B220 for B cells. Plus, the hematopoietic lineage eFluor450® Cocktail, PE-Sca-1, APC-c-Kit, Percp-cy5.5-CD150, APC-Cy7-CD48, PE-Cy7-CD16/32 and Alexa Fluor700-CD34 for HSCs and progenitors. A LSC cell cycle analysis was performed by staining cells with Hoechst 33342 at 37 °C for 90 min. After staining, the cells were washed once with PBS and analyzed using a BD LSR Fortessa flow cytometer. The FACS data were analyzed and represented using Flowjo10 software.

### H&E staining of histology

The spleen, lung or tibia from model mice were fixed in 4% Paraformaldehyde for 24 h at room temperature, followed by an overnight rinse in water. Sections were stained with H&E using routine methods. All images were obtained using a digital pathology scanner (3D HISTECH) and imported into Case Viewer software as a series of tagged image files. The representative pictures were taken at a 20x, 50x, or 100x magnification, the scale bars correspond to 200 μm or 500 μm.

### Western blotting and Immunoprecipitation

Cells were lysed with RIPA buffer containing protease and phosphatase inhibitors. Proteins were separated using PAGE gel electrophoresis, transferred onto PVDF membranes and immunoblotted with primary antibodies, followed by an incubation with horseradish peroxidase-conjugated secondary antibodies. The final signal was detected with enhanced chemiluminescence (ECL). The primary antibodies included: HA (CST, #3724), TSPAN32 (Santa, sc-81997), TAL1 (Huabio, RT1605), ABL (Santa, Sc-23), p-AKT (CST, #4060), AKT (CST, #9272), PTEN (CST, #9188) and ACTIN (Santa, sc-8432). For the immunoprecipitation assay, whole cell extracts were prepared with lysis buffer (P0013; Beyotime) and incubated with the appropriate antibody overnight at 4 °C. Protein A&G beads (Santa, sc-2003) were added, incubated for 4 h at 4 °C and washed three times with PBS buffer. The bound proteins were then separated using SDS-PAGE, transferred to a PVDF membrane and probed with the appropriate antibodies. The final signal was visualized using an ECL analyzer. The immunoprecipitation samples were also subjected to SDS-PAGE enrichment for a mass spectrometry analysis, which was completed by OE-biotech (Shanghai, China).

### Statistical analysis

The statistical significance of the differences between the experimental and control groups were analyzed using a student’s *T*-test with GraphPad Prism software (version 7.01 for Windows). The Kaplan–Meier method was adopted for animal survival analysis to generate survival curve; the significance of the differences was determined using a log-rank analysis. *P* = 0.05 was used as the threshold for determining the statistical significance of the data, which was further indicated using the following standard notation: no significance (N.S.), *P* ≤ 0.05 (*), *P* ≤ 0.01 (**) and *P* ≤ 0.001 (***).

## Supplementary information

Supplementary Materials

## Data Availability

The data and materials used in the current study are available from the corresponding authors upon reasonable request.
