# Supplementary material for: TSPAN32 suppresses chronic myeloid leukemia pathogenesis and progression by stabilizing PTEN
Source: Signal Transduct Target Ther. 2023 Mar 1;8:90. doi: 10.1038/s41392-022-01290-7 (PMC9974991; doi:10.1038/s41392-022-01290-7)
Supplement: Supplementary file 1 — Supplementary Materials [file 41392_2022_1290_MOESM1_ESM.docx]

Supplementary Materials for

**TSPAN32 suppresses chronic myeloid leukemia pathogenesis and progression by stabilizing PTEN**

Qiang Qiu^1,2#^, Yuanyuan Sun^1,2#^, Linyu Yang^2#^, Qingqing Li^1,2^, Yunyu Feng^1,2^, Mengyuan Li^1,2^, Yuexia Yin^1,2^, Li Zheng^1,2^, Ning Li^1,2^, Huandi Qiu^1,2^, Xue Cui^1,2^, Wei He^1,2^, Bochuang Wang^1,2^, Cong Pan^1,2^, Zi Wang^3^, Juan Huang^4^, Klarke M. Sample^5^, Zhihui Li^6,7^, Yiguo Hu^1,2,7*^

^1^ Department of Thyroid Surgery, National Clinical Research Center for Geriatrics, West China Hospital, Sichuan University, Chengdu, Sichuan, China.

^2^ State Key Laboratory of Biotherapy and Cancer Center, West China Hospital, Sichuan University, and Collaborative Innovation Center for Biotherapy, Chengdu, Sichuan, China.

^3^ Department of Oncology, Guizhou Provincial People's Hospital, Guiyang, Guizhou, China.

^4^ Sichuan Provincial People's Hospital, Chengdu, Sichuan, China.

^5^ Institute of Life Science, eBond Pharmaceutical Technology Ltd., Chengdu, China.

^6^ Laboratory of thyroid and parathyroid disease, Frontiers Science Center for Disease-related Molecular Network, West China Hospital, Sichuan University, Chengdu, Sichuan, China.

^7^ Department of Thyroid Surgery, West China Hospital, Sichuan University, Chengdu, Sichuan, China.

# These authors contributed equally to this work.

^*^ Correspondence to: Yiguo Hu (huyiguo@scu.edu.cn) West China Hospital, Sichuan University, Chengdu 610041, Sichuan, China; Phone: 8628-8516-4063; Fax: 8628-8516-4060.

**This PDF file includes:**

Figures. S1 to S3


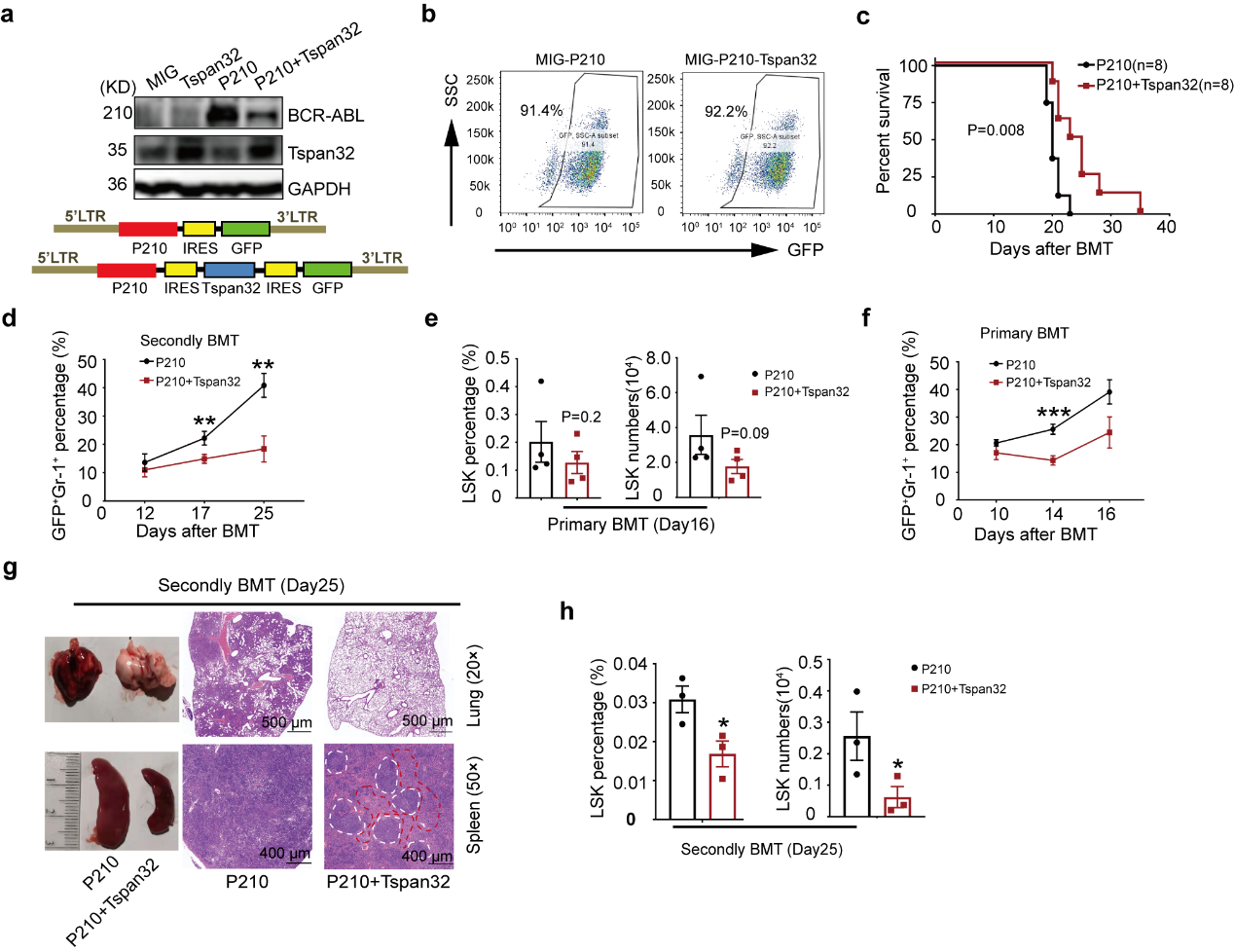


**Figure. S1.**

**Tspan32 overexpression prolongs the survival of BCR-ABL induced CML mice.** (**a**) Construction of BCR-ABL and Tspan32 coexpression vector (MSCV-P210-IRES-Tspan32-IRES-GFP), immunoblotting for BCR-ABL, TSPAN32 expression in HEK-293T cells, GAPDH served as the loading control. (**b**) The viral titer obtained from the 3T3 cells was determined using the percentage of GFP positive cells. (**c**) Recipient mouse Kaplan-Meier-style survival curves for P210 or P210-TSPAN32 transduced BM cells from WT mice. (**d**) Percentage of leukemia cells were monitored in the recipient PB on days 10, 14 and 16 post BMT. (**e**) The percentage and number of LSCs in the recipient BM per group were analyzed on day 16 (n=4 per group). (**f**) Secondary BMT was performed on day 16, the percentages of leukemia cells in PB were analyzed for all recipients on day 12, 17 and 25 post-secondary BMT. (**g-h**) On day 25 after secondary BMT, the recipients were sacrificed, gross appearance and H&E-staining for spleen and lung were analyzed, the white dotted line represents the white pulp of spleen, and red dotted line represents the red pulp of spleen. The percentage and number of LSCs in the BM of two groups were analyzed (n=3 per group). Error bars denote mean ± SEM, *P<0.05, **P<0.01, ***P<0.001 (T-test).


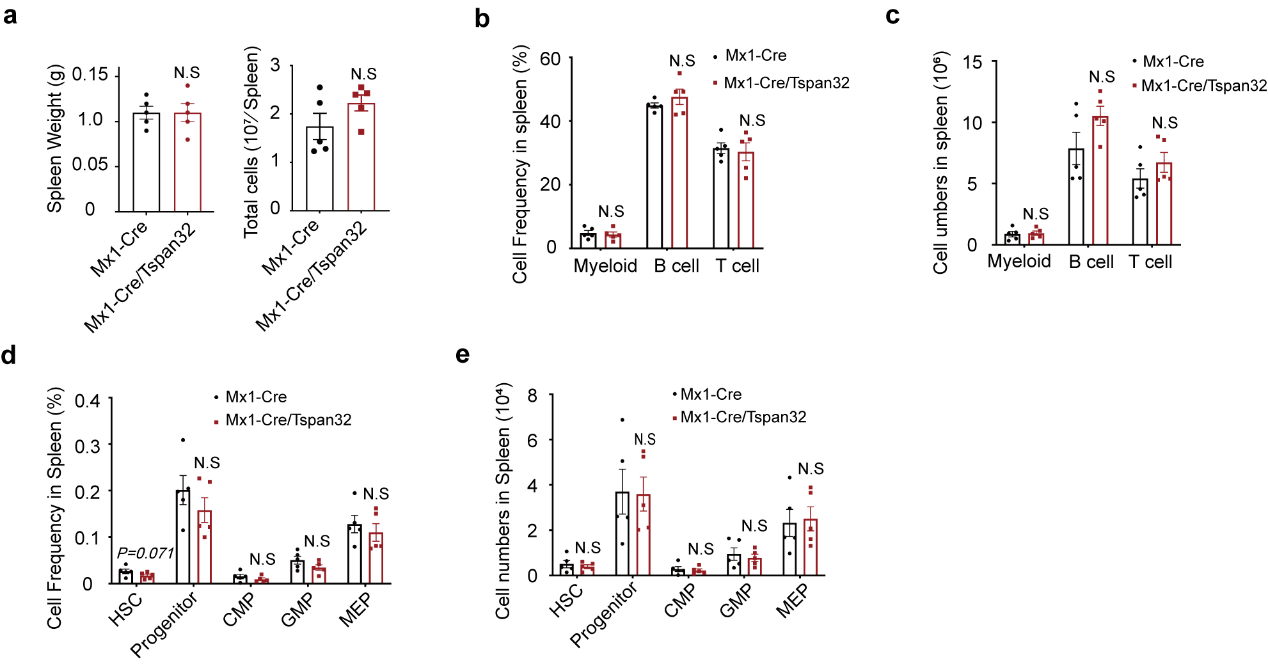


**Figure. S2.**

**The effect of Tspan32 on the normal hematopoietic system.** Mx1-Cre/Tsp-KI mice were treated four times with 15 mg/kg Pipc every other day. HSCs and hematopoiesis were analyzed after 30 days. (**a**) Spleen weight and total cell number are displayed. (**b-c**) The percentage and number for the different lineages including myeloid cell (Gr-1), B cell and T cell in spleen were summarized. (**d-e**) The percentage and number of HSCs and progenitors (including CMP, GMP, MEP) in the spleen were analyzed and shown (n=5 per group). Error bars denote mean ± SEM, N.S, no significance (T-test).


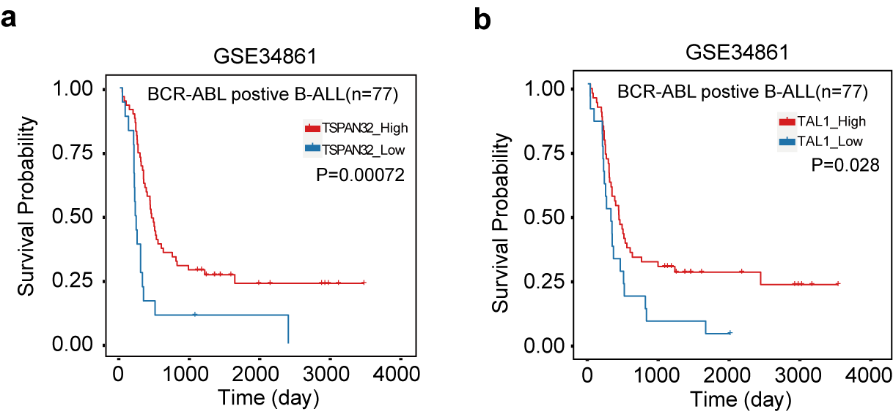


**Figure. S3.**

**High-expression of TSPAN32/TAL1 is associated with favorable probability of overall survival in *BCR-ABL*^+^ B-ALL patients.** **(a)** Dataset of GSE76312, based on low or high expression of TSPAN32, Kaplan-Meier plots depiction the probability of overall survival (OS) in BCR-ABL^+^ B-ALL patients (n=77, Log-rank test). **(b)** Based on low or high expression of TAL1, Kaplan-Meier plots depiction the probability of overall survival (OS) in BCR-ABL^+^ B-ALL patients (n=77, Log-rank test).
